# Supplementary material for: MetaRibo-Seq measures translation in microbiomes
Source: Nat Commun. 2020 Jun 29;11:3268. doi: 10.1038/s41467-020-17081-z (PMC7324362; doi:10.1038/s41467-020-17081-z)
Supplement: Supplementary file 10 — Supplementary Data 7 [file 41467_2020_17081_MOESM10_ESM.zip › File2/Confidence_High_Taxonomy/433161_out.krona.html]

Javascript must be enabled to view this page.

members
magnitude
magnitudeUnassigned
count
unassigned
taxon
rank

433161\_out

14

2
14
superkingdom

976
14
phylum

200643
class
14

171549
14
order

14
family
815

5
816

SRS017247\_contig\_number\_8566SRS063813\_contig\_number\_4788SRS075021\_contig\_number\_3101SRS1041112\_contig\_number\_5362SRS147088\_contig\_number\_contig-100\_439.52265
genus
14

28116

SRS015694\_contig\_number\_22577SRS019496\_contig\_number\_4216SRS024009\_contig\_number\_9408SRS024075\_contig\_number\_9509SRS062654\_contig\_number\_23125SRS098644\_contig\_number\_43476SRS104693\_contig\_number\_8536SRS147022\_contig\_number\_9834
species
8

371601
1
species

SRS011405\_contig\_number\_14255
